# Cholesterol Accumulation as a Driver of Hepatic Inflammation Under Translational Dietary Conditions Can Be Attenuated by a Multicomponent Medicine

**DOI:** 10.3389/fendo.2021.601160

**Published:** 2021-03-18

**Authors:** Andrea M. Mueller, Robert Kleemann, Eveline Gart, Wim van Duyvenvoorde, Lars Verschuren, Martien Caspers, Aswin Menke, Natascha Krömmelbein, Kanita Salic, Yvonne Burmeister, Bernd Seilheimer, Martine C. Morrison

**Affiliations:** ^1^ Systems Research and Development, Heel GmbH, Baden-Baden, Germany; ^2^ Department of Metabolic Health Research, The Netherlands Organisation for Applied Scientific Research (TNO), Leiden, Netherlands; ^3^ Department of Vascular Surgery, Leiden University Medical Center, Leiden, Netherlands; ^4^ Human and Animal Physiology, Wageningen University, Wageningen, Netherlands; ^5^ Department of Microbiology and Systems Biology, The Netherlands Organisation for Applied Scientific Research (TNO), Leiden, Netherlands

**Keywords:** non-alcoholic fatty liver disease, obesity, cholesterol, inflammation, diet-induced, liver, neutrophils, hepar compositum

## Abstract

**Background:**

Non-alcoholic fatty liver disease (NAFLD) is a complex multifactorial disorder that is characterised by dysfunctional lipid metabolism and cholesterol homeostasis, and a related chronic inflammatory response. NAFLD has become the most common cause of chronic liver disease in many countries, and its prevalence continues to rise in parallel with increasing rates of obesity. Here, we evaluated the putative NAFLD-attenuating effects of a multicomponent medicine consisting of 24 natural ingredients: Hepar compositum (HC-24).

**Methods:**

Ldlr-/-.Leiden mice were fed a high-fat diet (HFD) with a macronutrient composition and cholesterol content comparable to human diets for 24 weeks to induce obesity-associated metabolic dysfunction, including hepatic steatosis and inflammation. HC-24 or vehicle control was administered intraperitoneally 3 times/week (1.5 ml/kg) for the last 18 weeks of the study. Histological analyses of liver and adipose tissue were combined with extensive hepatic transcriptomics analysis. Transcriptomics results were further substantiated with ELISA, immunohistochemical and liver lipid analyses.

**Results:**

HFD feeding induced obesity and metabolic dysfunction including adipose tissue inflammation and increased gut permeability. In the liver, HFD-feeding resulted in a disturbance of cholesterol homeostasis and an associated inflammatory response. HC-24 did not affect body weight, metabolic risk factors, adipose tissue inflammation or gut permeability. While HC-24 did not alter total liver steatosis, there was a pronounced reduction in lobular inflammation in HC-24-treated animals, which was associated with modulation of genes and proteins involved in inflammation (e.g., neutrophil chemokine Cxcl1) and cholesterol homeostasis (i.e., predicted effect on ‘cholesterol’ as an upstream regulator, based on gene expression changes associated with cholesterol handling). These effects were confirmed by CXCL1 ELISA, immunohistochemical staining of neutrophils and biochemical analysis of hepatic free cholesterol content. Intrahepatic free cholesterol levels were found to correlate significantly with the number of inflammatory aggregates in the liver, thereby providing a potential rationale for the observed anti-inflammatory effects of HC-24.

**Conclusions:**

Free cholesterol accumulates in the liver of Ldlr-/-.Leiden mice under physiologically translational dietary conditions, and this is associated with the development of hepatic inflammation. The multicomponent medicine HC-24 reduces accumulation of free cholesterol and has molecular and cellular anti-inflammatory effects in the liver.

**Graphical Abstract:**
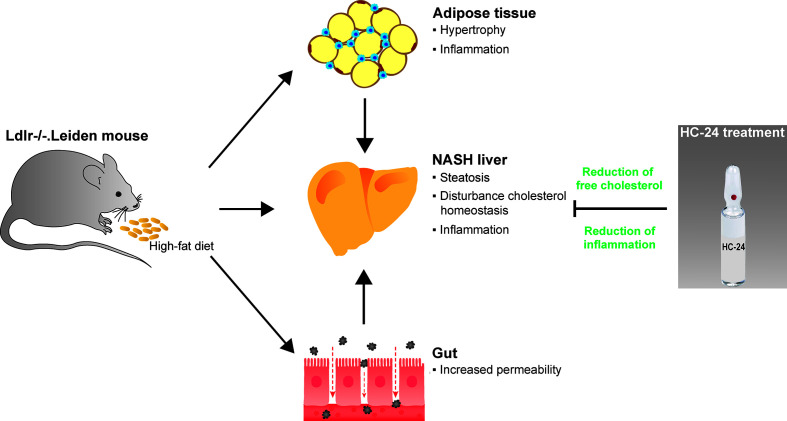
High-fat diet feeding in Ldlr-/-. Leiden mice induces obesity and associated metabolic dysfunction in adipose tissue (hypertrophy and inflammation), gut (increased permeability) and liver (non-alcoholic steatohepatitis; NASH). This NASH development in the liver is characterised by steatosis with disturbed cholesterol homeostasis and an associated inflammatory response, i.e. the infiltration of immune cells such as macrophages and neutrophils. Treatment with the multicomponent medicinal product HC-24 reduces hepatic accumulation of the cytotoxic lipid species free cholesterol and has anti-inflammatory effects in the liver – specifically reducing the infiltration of neutrophils into this tissue.

## Introduction

Non-alcoholic fatty liver disease (NAFLD) encompasses a spectrum of chronic liver disease which ranges from simple steatosis to non-alcoholic steatohepatitis (NASH), the progressive form of the disease which is characterised by hepatic inflammation and fibrosis in addition to steatosis. With a global prevalence of 25%, NAFLD is set to become the most common form of chronic liver disease worldwide (1). It is estimated that this prevalence will continue to increase, along with increasing rates of obesity and type 2 diabetes (2). As a result, NAFLD is projected to become the leading indication for liver transplantation within the next decade (3), especially since there is currently no approved pharmacological therapy for NAFLD. Therefore, there is an immense unmet need for efficacious therapeutics for NAFLD.

It is well accepted that the pathogenesis of NAFLD is multifactorial and also involves metabolic dysfunction in extrahepatic tissues such as the adipose tissue and the gut (4). In the liver, many molecular pathways contribute to the development and progression of the disease (5). An important factor that is thought to drive NAFLD progression from simple steatosis to steatohepatitis is cholesterol (6). It is becoming increasingly clear that accumulation of cholesterol in the liver (especially in its free, unesterified form) is a potent inducer of hepatic inflammation during NAFLD development (7, 8). In NAFLD patients, free cholesterol levels in the liver are associated with disease severity (9, 10), and many rodent models for NASH require dietary cholesterol supplementation to induce liver inflammation [reviewed in (11)]. It remains unclear, however, whether the disturbance of cholesterol homeostasis and the associated inflammatory response observed in humans is replicated in rodent models for NAFLD that use more human-like dietary conditions (i.e., diets with a macronutrient composition and a natural cholesterol content similar to human diets).

Given the multifactorial nature of the disease, it is conceivable that a successful therapy for NAFLD will need a multitarget therapy that targets several aspects of pathogenesis. Hepar compositum (HC-24) is a multicomponent medicinal product consisting of 24 ingredients including plant extracts, bioactive metabolites and animal-derived extracts (full composition shown in **Supplemental Table 1**). It has a long tradition of use as a supportive treatment for hepatic disorders of various origins (12), and there is anecdotal evidence for an effect of HC-24 on cholesterol metabolism (13), which possibly allows modulation of hepatic inflammatory responses. Many components of HC-24 have reported metabolic or anti-inflammatory effects. For instance, *Silybum marianum* (milk thistle) and its main constituent silymarin (rich in flavolignans) (14) have been shown to reduce plasma and liver markers of inflammation in high-fat diet (HFD)-fed mice (15). Similarly, *Taraxacum officinale* (dandelion) extracts have been shown to reduce LPS-induced inflammation in endothelial cells (16) and macrophages (17) and have cholesterol-modulating effects in rodents (18, 19) and type 2 diabetic patients (20). Likewise, *Avena sativa* (oat) is well-known for its anti-inflammatory and its hypocholesterolaemic effects (21).

Since both cholesterol metabolism and the inflammatory response are important interconnected physiological processes that are dysregulated during NAFLD development (8, 22), we questioned whether HC-24 might have beneficial effects in the treatment of NAFLD/NASH. To address this, we investigated the potential metabolic and anti-inflammatory effects of HC-24 in a translational diet-induced mouse model of NAFLD, the Ldlr-/-.Leiden mouse. In response to a HFD with a macronutrient composition and cholesterol content that is comparable to human diets (23–25), Ldlr-/-.Leiden mice develop histopathological characteristics of human NAFLD in the context of an obese phenotype with dyslipidaemia, hypertriglyceridaemia and insulin resistance (26–28) as is typical for a large proportion of NAFLD patients (29–31). In addition, many molecular processes that underlie the pathogenesis in NAFLD patients (both on the level of liver transcriptomics and of plasma metabolomics) are also reflected in the Ldlr-/-.Leiden mouse (32–34). Use of the aforementioned HFD with a translational cholesterol content enabled us to study the potential build-up of cholesterol in livers under physiologically relevant dietary conditions and to investigate the effects of HC-24 on the development of NAFLD.

## Materials and Methods

### Animal Study

The animal experiment was performed in the AAALAC-accredited animal facility at TNO Metabolic Health Research (Leiden, the Netherlands) in accordance with the rules and regulations set forward by the Netherlands Law on Animal Experiments with ethical approval by an independent Animal Welfare Body (IvD TNO; approval number 3682/TNO-210). Animals were group-housed in makrolon cages (2–4 mice/cage) in animal rooms with 50-60% relative humidity, temperature ~21°C and a 7 am to 7 pm light cycle with *ad libitum* access to food and water. Male Ldlr-/-.Leiden mice (age 13–16 weeks) were obtained from the breeding colony at TNO Metabolic Health Research (Leiden, the Netherlands). All animals were kept on a standard rodent maintenance diet until the start of the study (9 kcal% fat, 33 kcal% protein, 58 kcal% carbohydrate; R/M-H, Ssniff Spezialdiäten, Soest, Germany). At the start of the study, one group of mice (chow, n=10) was kept on this maintenance diet as a reference group. The rest of the mice (n=30) were switched to an energy-dense high-fat diet (HFD; 45 kcal% fat from lard, 20 kcal% protein, 35 kcal% carbohydrate; D12451, Research diets, New Brunswick, NJ, USA) to induce obesity, metabolic dysfunction and NAFLD. This diet is characterised by a macronutrient composition that mimics human diets (23–25), and the natural cholesterol content of this diet (approx. 0.02% w/w) was not further increased by addition of supraphysiological amounts of cholesterol. After 6 weeks of HFD feeding, these mice were matched into 2 groups based on body weight, blood glucose and plasma cholesterol levels. From that point onwards, the HFD+vehicle control group was treated with vehicle (saline), and the HFD+HC-24 group was treated with HC-24 (Heel GmbH, Baden-Baden, Germany; full composition shown in **Supplemental Table 1**). Both treatments were provided by intraperitoneal injection 3x weekly (Monday, Wednesday and Friday) at a dose of 1.5 ml/kg body weight. Body weight and food intake were monitored throughout the study. Body composition (lean mass and fat mass, expressed as % of body weight) was determined in the last week of the study (t=24 weeks) by EchoMRI (EchoMRI-LLC, Houston, TX, USA). Fasting (5 h) blood samples for EDTA plasma isolation were collected via the tail vein. *In vivo* gut permeability was assessed in week 23 using an FD4 gut permeability assay as described previously (28). In short, mice were fasted for 5 h after which a baseline blood sample was taken *via* the tail vein. Then, FITC-labeled dextran (three to five kDa FD4; Sigma Aldrich, St. Louis, MO, USA) was administered by oral gavage (900 mg/kg) and 4 h later a second blood sample was collected to determine the plasma FD4 concentration using a fluorometer (FLUOstar Galaxy, BMG labtech, Offenburg, Germany). The fluorescence reading was corrected for autofluorescence using the baseline blood sample. There was no drop out of mice during the study. All mice were terminated (unfasted) by gradual-fill CO_2_ asphyxiation after 24 weeks of chow/HFD feeding. A terminal blood sample for EDTA plasma was collected by cardiac puncture. The epididymal and mesenteric white adipose tissue depots were isolated and weighed and then fixed in formalin and embedded in paraffin for histological analyses. Livers were isolated and weighed, the medial lobe was fixed in formalin and embedded in paraffin for histological analyses, the left lobe was snap-frozen in liquid N_2_ and stored at -80°C for biochemical analyses.

### Plasma Biochemistry

Blood glucose was determined during blood sampling using a hand-held glucometer (Freestyle Freedom Light, Abbott Laboratories, Lake Bluff, IL, USA). Plasma insulin was determined by ELISA (Chrystal Chem Inc., Downers Grove, IL, USA). Plasma lipids (total cholesterol and triglycerides) were assayed in freshly prepared EDTA-plasma using commercially available enzymatic assays (CHOD-PAP and GPO-PAP respectively; Roche Diagnostics, Almere, the Netherlands). Plasma serum amyloid A (SAA) was measured by ELISA (Invitrogen/Thermo Fisher Waltham, MA, USA). Plasma alanine aminotransferase (ALT) was assessed using a spectrophotometric activity assay (Reflotron-plus, Roche Diagnostics). All these analyses were performed according to the manufacturers’ instructions.

### Histological Analysis of Adipose Tissue and Liver

Paraffin-embedded cross-sections (5 μm) of the epididymal and the mesenteric white adipose tissue were stained with hematoxylin-phloxine-saffron and digitised using a slide scanner (Aperio AT2, Leica Biosystems, Amsterdam, the Netherlands). Adipose tissue morphometry (average adipocyte size and adipocyte size distribution) and inflammation (number of crown-like structures; CLS per 1000 adipocytes) were analysed as described previously (35), using the automated image analysis software Adiposoft for morphometry analyses (36) and counting the number of CLS in the same fields used for morphometry analyses. Formalin-fixed and paraffin-embedded cross-sections of the medial liver lobe (3 μm) were stained with haematoxylin and eosin and scored blindly by a board-certified pathologist using an adapted grading method for human NASH (37, 38) as described previously (39). Briefly, two cross-sections/mouse were examined and the level of microvesicular steatosis, macrovesicular steatosis and hepatocellular hypertrophy were determined relative to the liver area analysed (expressed as a percentage). Hepatic inflammation was assessed by counting the number of inflammatory foci per field at a 100× magnification in five non-overlapping fields per specimen, expressed as the number of foci per mm^2^. Analysis of hepatic neutrophil counts was performed by immunohistochemical staining for GR-1 (with FITC-conjugated rat anti-mouse monoclonal antibody 1:1,000, eBioscience/Thermo Fisher; followed by rabbit anti-FITC recombinant monoclonal antibody 1:1,000, Invitrogen/Thermo Fisher; and BrightVision Poly-HRP goat anti-rabbit antibody 1:1, VWR International B.V., Amsterdam, the Netherlands) after enzymatic antigen retrieval using 0.025% pepsin (Sigma Aldrich) in 0.01 M HCl for 15 min at 37°C. Sections were stained with ImmPACT NovaRED HRP substrate (Vector Laboratories, Burlingame, CA, USA) and counterstained with haematoxylin. Neutrophils were quantified by counting the number of GR-1-positive inflammatory foci per field at 100× magnification in five non-overlapping fields per specimen, expressed as the number of positive foci per mm^2^. Analysis of hepatic macrophages was performed by immunohistochemical staining for F4/80 (rat anti-mouse monoclonal antibody 1:500, eBioscience; followed by biotinylated goat anti-rat secondary antibody 1:300, Abcam, Cambridge, UK) after heat-mediated antigen retrieval in sodium citrate buffer pH 6 in a PT link system (both DAKO/Agilent, Amstelveen, the Netherlands) and endogenous peroxidase block with 0.3% H_2_O_2_ in methanol. Sections were stained using Vectastain ABC-HRP Kit and DAB peroxidase substrate (both Vector Laboratories) and counterstained with haematoxylin. Macrophages were quantified by counting the number of F4/80-positive crown-like structures per field at 100× magnification in five non-overlapping fields per specimen, expressed as the number of CLS per mm^2^ as described previously (33).

### Liver Transcriptome Analysis

RNA was isolated from snap-frozen liver tissue samples (left lobe) from all mice using RNA-Bee Total-RNA Isolation Kit (Bio-Connect, Huissen, the Netherlands). RNA concentration was determined spectrophotometrically using Nanodrop 1000 (Isogen Life Science, De Meern, the Netherlands) and RNA quality was assessed using a 2100 Bioanalyzer (Agilent Technologies, Amstelveen, the Netherlands). Then, next-generation RNA sequencing was performed on livers from all mice. For this, RNA was used to generate strand-specific cDNA libraries for Next Generation Sequencing according to the manufacturer’s protocol by GenomeScan B.V. (Leiden, the Netherlands; using oligo-dT magnetic beads, mRNA fragmentation, NEBNext Ultra Directional RNA Library Prep Kit from Illumina, NEB #E7420S/L resulting in 300–500 bp amplified libraries/sample). Libraries were multiplexed, clustered, and sequenced on a NextSeq500 system (Illumina, San Diego, CA, USA, using 1.6 pM of cDNA/sample and NextSeq control software version 2.0.2) with a single-read 75 cycles sequencing protocol, 12 million reads per sample and indexing. Image analysis, base calling, and quality check was performed with the Illumina data analysis pipeline RTA v2.4.11 and Bcl2fastq v2.17. Three mice were excluded as biological outliers (described below under statistical analysis), and one mouse (from the HFD + vehicle group) was excluded as a technical outlier (based on principal component analysis of the sequencing data), resulting in: n=10 chow, n=13 HFD+vehicle, n=13 HFD+HC-24 for further statistical analysis of the sequencing data. The gene expression data is publicly available via the NCBI Gene Expression Omnibus (GEO) database (https://www.ncbi.nlm.nih.gov/geo/under accession number GSE163652). The number of differentially expressed genes (DEG) between groups was determined using an established statistical analysis procedure [DEseq2 pipeline (40)]. These DEG were determined using a statistical cut-off of p<0.01 for the following two comparisons: HFD+vehicle vs chow; and HFD+HC-24 vs HFD+vehicle. Next, these DEG were used as an input for an upstream regulator analysis through Ingenuity Pathway Analysis suite (IPA; www.ingenuity.com, accessed 2017). This analysis integrates the expression of a multitude of genes downstream from predefined upstream regulators (e.g., transcription factors, signalling proteins, metabolites) thus allowing summation of multiple (small) gene expression changes to provide information on the predicted activation state of such an upstream regulator. A negative Z-score <-2 indicates a predicted reduction in activity based on the direction of gene expression changes of target genes. A positive Z-score >2 indicates activation of the upstream regulator.

### Quantification of CXCL1 in Liver Tissue

Liver homogenates were prepared from the sinister lobe in ice-cold lysis buffer (50 mM Tris-HCl, 150 mM NaCl, 5mM CaCl_2_, 1% Triton X-100, pH 7.4) supplemented with complete mini protease inhibitor cocktail (Roche). Homogenates were centrifuged and the supernatant was used for CXCL1 determination by ELISA (Mouse CXCL1/KC Quantikine ELISA, R&D systems, Abingdon, UK) according to the manufacturer’s instructions. CXCL1 levels were expressed per mg liver protein, as determined by bicinchoninic acid (BCA) assay (Pierce/Thermo Fisher) in the same homogenates used for CXCL1 analysis.

### Biochemical Analysis of Liver Lipids

Intrahepatic levels of triglycerides, cholesteryl esters and free cholesterol were determined in freshly prepared liver homogenates by high-performance thin-layer chromatography (HPTLC). Lipids were extracted from liver homogenates using methanol and chloroform as described previously (41, 42). After extraction, lipids were separated by high-performance thin-layer chromatography on silica gel 60 plates (0.20 mm). Lipid spots were stained with colour reagent (5 g of MnCl_2_∙4H_2_O, 32 ml of 95–97% H_2_SO_4_ added to 960 ml of CH_3_OH/H_2_O) and triglycerides, cholesteryl esters and free cholesterol were quantified using a Chemidoc Touch imaging system and Image Lab software (both Bio Rad Laboratories, Veenendaal, the Netherlands). Liver lipids were expressed as µg lipid per mg liver tissue.

### Cholesterol Balance Analysis

Whole-body cholesterol balance (i.e., determination of net production or net excretion of cholesterol) was assessed by combining dietary cholesterol intake data with faecal cholesterol excretion data. In week 16 and week 20 of the study, faeces were collected from each cage over a period of 3 or 4 days respectively. Food intake was measured over the same period and dietary cholesterol intake was determined. Faecal neutral sterol and faecal bile acid levels were assessed to determine faecal excretion of cholesterol as described previously (43, 44). Faecal samples were first lyophilized and weighed. For extraction of bile acids, a 5 mg aliquot of faeces was incubated in 1 ml alkaline methanol (3:1 v/v) for 2 h at 80˚C, using nor-hyodeoxycholate as an internal standard. Samples were then diluted in distilled water, mixed and centrifuged. The supernatant was applied to a prepared Sep-Pak C18 solid-phase extraction cartridge (Waters Corporation, Wexford, Ireland) and bile acids were eluted with 100% methanol. Bile acids were derivatized by incubation with trifluoroacetic anhydride and 1,1,1,3,3,3-hexafluoro-2-propanol for 1 h at 60˚C after which they were separated using a 25 m × 0.25 mm capillary gas chromatography column (CP-Sil 5B, Agilent, Santa Clara, CA, USA; temperature programmed from 230°C to 280°C) in a Scion 436-GC gas chromatography system (Scion Instruments, Livingstone, UK) equipped with a flame ionization detector (kept at 300°C). Bile acid derivatives were introduced by split injection (split ratio, 20:1; injector temp 300°C). Quantitation of bile acids (cholic acid, deoxycholic acid, litocholic acid, α-muricholic acid, β-muricholic acid, ω-muricholic acid, and hyodeoxycholic acid) was based on the area ratio of the individual bile acid to the internal standard. Levels of α-muricholic acid were below the lower limit of quantitation and are therefore not reported. For extraction of neutral sterols, a 10 mg aliquot of faeces was incubated in 1 ml alkaline methanol as described for bile acid extraction, using 5α-cholestane as an internal standard. Next, neutral sterols were extracted three times with petroleum ether. The combined petroleum ether layers were then evaporated and the neutral sterols were silylated with DMF Sil-prep after which they were separated by GC using the same column and protocol as for the bile acids. Quantitation of neutral sterols (coprostanol, cholesterol, cholestanol, and lathosterol) was based on the area ratio of the individual neutral sterol to the internal standard. The data from the food intake measurements and the sterol and bile acid excretion analyses was used to calculate net cholesterol production as follows: cholesterol excretion (i.e., bile acid + neutral sterol excretion) – dietary cholesterol intake, expressed as µmol/mouse/day.

### Statistical Analysis

With the exception of the transcriptomics analysis, for which the statistical analysis has been described above, statistical analyses were performed using SPSS Statistics 24.0 (IBM, Armonk, NY, USA). Based on the robust regression and outlier removal test [ROUT (45)] with Q set to 1%, 3 mice (1 in the HFD + vehicle group and 2 in the HFD + HC-24 group) were identified as biological outliers and excluded from statistical analysis. Normal distribution of variables was analysed with the Shapiro-Wilk test, assuming normality at p>0.05. To test equality of variances, Levene’s test of homogeneity of variances was used, assuming equal variances at p>0.05. For normally distributed variables with equal variances, differences between groups were analysed by one-way analysis of variance (ANOVA) followed by one-sided Dunnet’s post-hoc tests. For normally distributed variables with unequal variances, differences between groups were analysed by analysis of variance (Brown-Forsythe) and one-sided Dunnett’s T3 post-hoc tests. The non-parametric Kruskall-Wallis test followed by post-hoc with one-sided Mann-Whitney U tests was used to determine differences between groups for variables that were not normally distributed. Correlation analyses were performed by Spearman’s rank correlation analysis. P-values <0.05 were considered statistically significant. Data are represented as means ± SD.

## Results

### HC-24 Does Not Affect HFD-Induced Obesity, Hyperinsulinaemia, Dyslipidaemia, or Systemic Inflammatory Marker SAA

Ldlr-/-.Leiden mice were fed an energy-dense high-fat diet for 24 weeks to induce obesity, metabolic dysfunction and NAFLD. Relative to a chow-fed reference group, HFD feeding resulted in increased body weight and adiposity (reduced lean mass % and increased fat mass %; **Table 1**). HFD feeding did not affect blood glucose but did result in strongly increased plasma insulin levels (**Table 1**), indicative of reduced insulin sensitivity. Plasma lipids, both total cholesterol and triglycerides (**Table 1**), were strongly increased in HFD-fed mice. In addition, HFD feeding resulted in an increase in the systemic inflammation marker serum amyloid A (SAA; **Table 1**). Treatment with HC-24 from week 6 of HFD feeding until the end of the study did not affect the development of obesity and adiposity, food intake, hyperinsulinaemia, dyslipidaemia, or systemic inflammation (**Table 1**).

**Table 1:** Metabolic risk factors at t=24 weeks.

	Chow	HFD +vehicle	HFD +HC-24
Body weight (g)	35.7 ± 4.1***	49.9 ± 4.8	51.0 ± 3.7
Lean mass (%)	76.8 ± 6.6***	57.3 ± 4.1	55.3 ± 4.3
Fat mass (%)	23.2 ± 6.6***	42.7 ± 4.1	44.7 ± 4.3
Average food intake (kcal/mouse/day)	13.4 ± 0.8	13.4 ± 0.9	13.7 ± 1.0
Fasting blood glucose (mM)	8.0 ± 1.0	7.9 ± 1.0	8.2 ± 0.6
Fasting plasma insulin (ng/ml)	2.1 ± 1.0**	08.8 ± 6.3	10.9 ± 6.5
Fasting plasma cholesterol (mM)	7.3 ± 1.8***	28.8 ± 6.2	28.1 ± 6.0
Fasting plasma triglycerides (mM)	0.9 ± 0.5***	4.5 ± 1.5	04.2 ± 2.1
Plasma serum amyloid A (µg/ml)	3.7 ± 1.5***	14.1 ± 4.9	14.0 ± 4.8

Asterisks indicate significance of difference with HFD + vehicle control group (**p < 0.01, ***p < 0.001). Data shown as mean ± SD.

### Adipose Tissue Hypertrophy and Inflammation, and Gut Permeability Are Not Affected by HC-24

**Figure 1:**
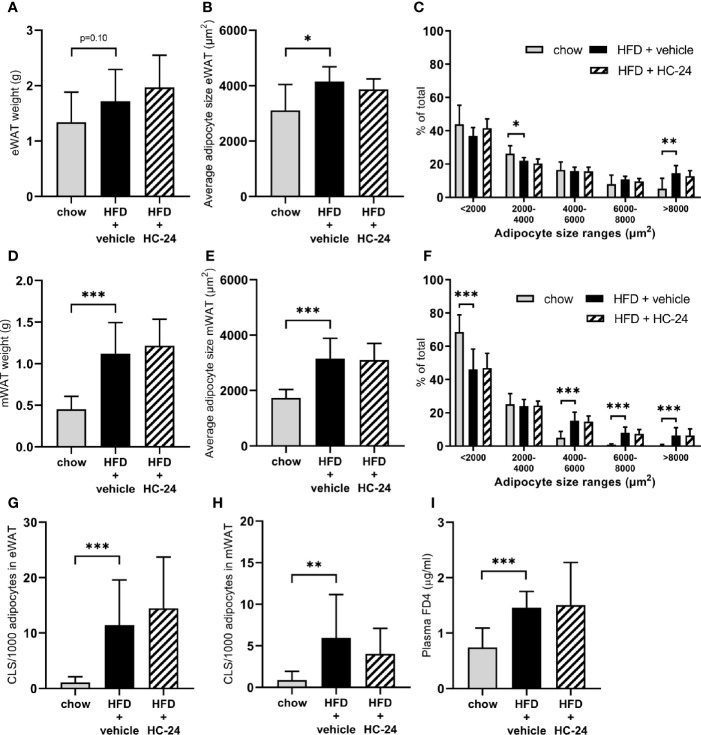
HC-24 does not affect adipose tissue mass, adipocyte hypertrophy or inflammation of epididymal and mesenteric white adipose tissue depots and has no effect on gut permeability in Ldlr-/-.Leiden mice fed a translational HFD for 24 weeks. **(A)** eWAT depot weight. **(B)** Average adipocyte size in eWAT. **(C)** Size distribution of adipocytes in eWAT. **(D)** mWAT depot weight. **(E)** Average adipocyte size in mWAT. **(F)** Size distribution of adipocytes in mWAT. **(G)** Number of CLS per 1,000 adipocytes in eWAT. **(H)** Number of CLS per 1000 adipocytes in mWAT. **(I)** *In vivo* functional gut permeability analysis using the Fluorescein isothiocyanate–labeled dextran (FD4) assay in week 23 of the study. eWAT, epididymal white adipose tissue; mWAT, mesenteric white adipose tissue; CLS, crown-like structure. Data are mean ± SD. *p < 0.05, **p < 0.01, ***p < 0.001 vs. HFD + vehicle.

In line with the observed effects on body weight and fat mass, HFD feeding tended to increase the weight of the epididymal white adipose (eWAT) depot and significantly increased the weight of the mesenteric white adipose (mWAT) depot (**Figures 1A, D**). This HFD-induced increase in adipose depot mass was accompanied by an increase in adipocyte hypertrophy in both depots, as shown by an increase in the average adipocyte size and a shift in the distribution of adipocyte sizes towards a reduction in the percentage of smaller adipocytes and an increase in the percentage of large adipocytes (**Figures 1B, C, E, F**). This expansion and hypertrophy of both eWAT and mWAT was associated with adipose tissue inflammation as shown by the increased number of crown-like structures (**Figures 1G, H**). HC-24 treatment did not affect adipose mass, hypertrophy or inflammation in either of the depots studied. Since increased gut permeability is thought to contribute to the progression of NAFLD, we investigated gut permeability with an FD4 test in week 23 of the study. HFD feeding significantly increased the passage of FD4 from the intestinal lumen into the circulation (**Figure 1I**), indicative of increased gut permeability. This was not affected by HC-24 (**Figure 1I**).

### HC-24 Does Not Affect Hepatic Steatosis But Does Significantly Reduce Hepatic Inflammation

**Figure 2:**
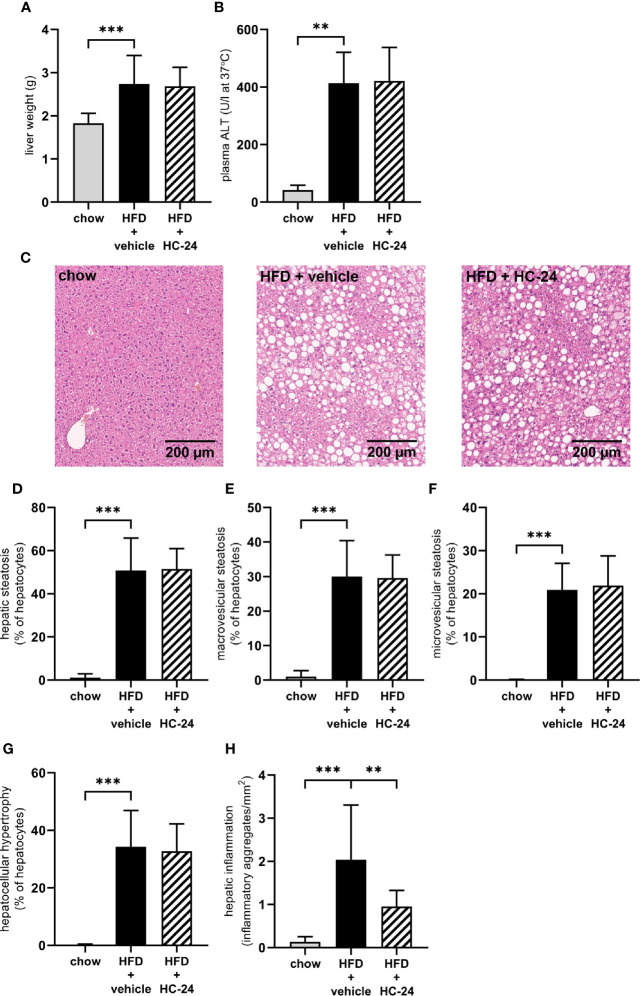
HC-24 treatment does not affect hepatic steatosis but does strongly reduce hepatic inflammation in Ldlr-/-.Leiden mice fed a translational HFD for 24 weeks. **(A)** Liver weight. **(B)** Plasma ALT measured in week 24 of the study. **(C)** Representative photomicrographs of Haemotoxylin and Eosin-stained cross sections of the medial lobe of the liver of chow, HFD + vehicle control and HFD + HC-24 groups. **(D)** Total steatosis. **(E)** Macrovesicular steatosis. **(F)** Microvesicular steatosis. **(G)** Hepatocellular hypertrophy. **(H)** Hepatic inflammation. HFD, high-fat diet; ALT, alanine aminotransferase. Data shown are mean ± SD. **p < 0.01, ***p < 0.001 compared with HFD + vehicle.

At the end of the 24-week study, liver weight was significantly increased in HFD-fed animals relative to chow (**Figure 2A**). This increase in liver weight was accompanied by an increase in plasma alanine aminotransferase (ALT; **Figure 2B**). Neither liver weight nor ALT were affected by HC-24 treatment. Histopathological analysis of NASH (representative images for all groups shown in **Figure 2C**) showed that HFD feeding resulted in pronounced hepatic steatosis (**Figure 2D**), which was roughly half in the macrovesicular form and half in the microvesicular form (**Figures 2E, F**). HC-24 treatment did not affect hepatic steatosis (**Figure 2D**) and did not affect the distribution of steatosis over the macrovesicular and the microvesicular form (**Figures 2E, F**). In line with the observations on hepatic steatosis, hepatocellular hypertrophy was increased by HFD-feeding, and was not affected by treatment with HC-24 (**Figure 2G**). However, quantitative analysis of the number of inflammatory aggregates in the liver showed a significant induction of hepatic inflammation in HFD-fed animals which was strongly and significantly reduced by treatment with HC-24 (**Figure 2H**). Altogether, this histopathological analysis of NASH shows a pronounced increase in steatosis and lobular inflammation in animals fed a HFD with a translational macronutrient composition and cholesterol content, and demonstrates a specific effect of HC-24 on the inflammatory component of the disease.

### HC-24 Modulates Genes Involved With Inflammation and Lipid Handling

To investigate the molecular processes that may underlie the observed anti-inflammatory effects of HC-24 in the liver, we performed a hepatic gene expression analysis using genome-wide RNA sequencing. This gene expression profiling was followed by an upstream regulator analysis which predicts the activation state of key regulatory factors (e.g., cytokines, transcription factors, metabolites) based on the expression pattern of genes downstream from this factor. As expected and in line with histopathologically observed changes in the liver, HFD feeding resulted in gene expression changes related among others to lipid metabolism and inflammation (modulated upstream regulators shown in **Supplemental Table 2**). The metabolite “cholesterol” was identified as a significantly modulated upstream factor (i.e., there was a predicted HFD-dependent increase in hepatic cholesterol based on gene expression patterns downstream from cholesterol). HC-24 treatment significantly affected the expression of distinct genes in the liver (full list shown in **Supplemental Table 3**). In line with the observed anti-inflammatory effect of HC-24 on the histological level, HC-24 modulated several inflammation-related genes, such as *Socs3* and *Gzma* and the neutrophil-attracting chemokine *Cxcl1*. Results from the upstream regulator analysis revealed further inflammation-modifying effects of HC-24, showing significant overlap with genes downstream of TGF-β, IL-17A, and IL-1β (**Supplemental Table 4**). Notably, this analysis also demonstrated significant inactivation of the upstream regulator cholesterol, suggesting reduced levels of cholesterol in the liver in HC-24 treated animals.

### HC-24 Lowers Liver CXCL1 Levels and Subsequent Neutrophil Infiltration Without Affecting Other Immune Cell Types

**Figure 3:**
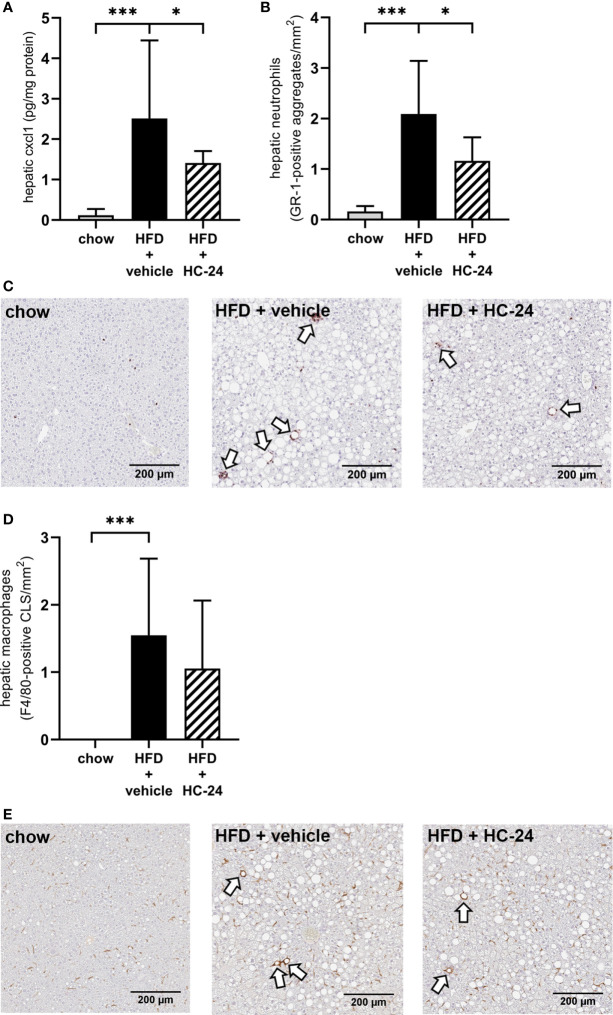
HC-24 treatment reduces neutrophil infiltration in Ldlr-/-.Leiden mice fed a translational HFD for 24 weeks. **(A)** Concentration of the neutrophil chemokine CXCL1 in liver tissue of chow, HFD + vehicle control and HFD + HC-24 groups. **(B)** Quantification of the immunohistochemical staining for the neutrophil marker GR-1, showing the number of GR-1-positive aggregates for each group. **(C)** Representative photomicrographs of immunohistochemical staining for the neutrophil marker GR-1 in cross sections of the medial lobe of the liver. Arrows indicate neutrophilic aggregates. **(D)** Quantification of the immunohistochemical staining for the macrophage marker F4/80, showing the number of F4/80-positive crown-like structures for each group. **(E)** Representative photomicrographs of immunohistochemical staining for the macrophage marker F4/80 in cross sections of the medial lobe of the liver. Arrows indicate F4/80-positive crown-like structures. HFD, high-fat diet; CLS, crown-like structure. Data shown are mean ± SD. *p < 0.05, ***p < 0.001 compared with HFD + vehicle.

To investigate whether the anti-inflammatory effect of HC-24 was indeed linked to an effect on neutrophil infiltration in the liver as suggested by the gene expression data, we measured CXCL1 protein levels in the liver (**Figure 3A**). In line with the observed modulation of *Cxcl1* gene expression, CXCL1 levels were low in chow-fed mice and significantly increased in HFD+vehicle controls. HC-24 treatment significantly lowered the levels of CXCL1 in liver tissue. Immunohistochemical staining of the neutrophil marker GR-1 followed by quantification of the number of GR-1-positive cell clusters in the liver (**Figure 3B**, with representative images shown in **Figure 3C**) further corroborated this effect: GR-1-positive cell aggregates were almost completely absent in chow controls, and their counts were significantly increased by HFD-feeding. Treatment with HC-24 significantly lowered the number of GR-1-positive cell aggregates in the liver, in line with the observed reduction in *Cxcl1* gene and protein expression. To explore whether HC-24 affects neutrophil infiltration exclusively or has a broader effect on immune cell infiltration in general, we analysed the transcriptome dataset using previously described immune cell-type specific gene sets that allow assessment of the presence of particular leukocytes in a tissue (46, 47). This analysis revealed that while there was an increased presence of B-cells and T-cells in HFD-fed mice relative to chow controls, this was not affected by HC-24 treatment (B-cells: HFD + vehicle vs. chow: −log(p-value) = 2.4, HFD + HC-24 vs HFD + vehicle: non-significant; T-cells: HFD + vehicle vs. chow: −log(p-value) = 2.3, HFD + HC-24 vs. HFD + vehicle: non-significant). In addition, we performed an immunohistochemical analysis of the macrophage marker F4/80, followed by quantification of F4/80-positive crown-like structures in liver (**Figure 3D**, with representative images shown in **Figure 3E**) to assess potential effects of HC-24 on this immune cell subset. Similar to what was observed for the B- and T-cell population, F4/80-positive crown like structures were significantly increased by HFD relative to chow, and this was not affected by treatment with HC-24.

### Biochemical Analysis of Liver Lipids Revealed an Effect of HC-24 on Hepatic Free Cholesterol Accumulation, Confirming the Observed Effects on the Gene Expression Level

**Figure 4:**
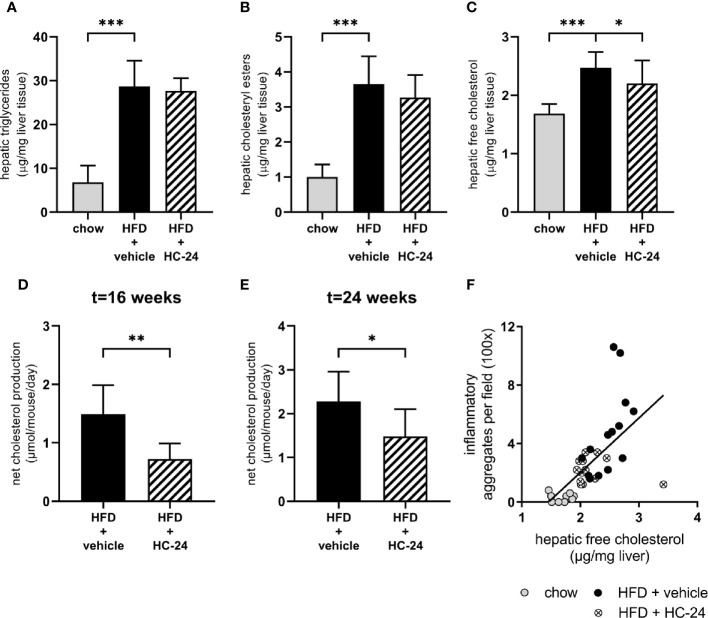
HC-24 treatment reduces free cholesterol accumulation in the liver of Ldlr-/-.Leiden mice fed a translational HFD for 24 weeks. **(A)** Hepatic triglyceride content. **(B)** Hepatic cholesteryl ester content. **(C)** Hepatic free cholesterol content. **(D)** Net whole-body cholesterol production in week 16 of the study. **(E)** Net whole-body cholesterol production in week 24 of the study. **(F)** Pearson correlation between free cholesterol in the liver and the histologically determined number of inflammatory aggregates in the liver. HFD, high-fat diet. Data shown are mean ± SD. *p < 0.05, **p < 0.01, ***p < 0.001 compared with HFD + vehicle.

Since free cholesterol is a potent inducer of hepatic inflammation and appeared to be critically affected by HFD feeding, we next investigated whether the predicted effect on cholesterol accumulation in the liver (based on observed gene signalling patterns downstream from cholesterol) may underlie the observed anti-inflammatory effects. For this, we first performed a biochemical analysis of intrahepatic lipids to confirm the predicted effect on hepatic cholesterol. This analysis showed that – like in NAFLD patients – triglycerides are the main lipid species that accumulate in the liver of HFD-fed mice (**Figure 4A**). In line with what was observed histologically, treatment with HC-24 did not affect the bulk storage of lipids as triglycerides (**Figure 4A**). In addition to triglycerides, cholesterol – both in the esterified form and in the free, unesterified form – also built up during NAFLD development, as can be observed in HFD-fed mice (**Figures 4B, C**). Treatment with HC-24 significantly reduced the build-up of free cholesterol (**Figure 4C**). To further substantiate this effect on hepatic cholesterol accumulation and to investigate how HC-24 affected hepatic free cholesterol levels, we investigated whether HC-24 had an effect on whole-body cholesterol balance. For this we measured faecal cholesterol excretion (in the form of neutral sterols and bile acids) and combined this with the dietary intake of cholesterol to calculate net excretion/production of cholesterol (**Figures 4D, E** and **Supplemental Table 5**). This analysis revealed that in HFD-fed Ldlr-/-.Leiden mice, there is a net production of cholesterol in the body (i.e., more cholesterol is excreted via the faeces than is consumed via the diet). Treatment with HC-24 significantly lowered the net production of cholesterol, in line with the effects on intrahepatic cholesterol accumulation. Furthermore, the levels of hepatic free cholesterol showed a significant positive correlation with hepatic inflammation (number of inflammatory cell foci; Spearman r = 0.78, p<0.001; **Figure 4F**), providing a rationale for the observed simultaneous effects of HC-24 on cholesterol homeostasis and liver inflammation.

## Discussion

This study shows that accumulation of free cholesterol in the liver, under translational dietary conditions, is associated with hepatic inflammation and can be modulated by a multicomponent medicinal product HC-24, resulting in reduced inflammatory aggregates with fewer neutrophils, a cell type that is characteristic for human NASH.

Many conventional rodent models for NAFLD rely on supplementation of diets with high (supraphysiological) levels of cholesterol to induce liver inflammation (48). While the accumulation of cholesterol *per se* is not irrelevant for NAFLD pathogenesis, since hepatic free cholesterol is also increased and associated with disease severity in NAFLD patients (9, 10), such models strongly emphasise the role of dietary cholesterol in NAFLD. Furthermore, they imply that an increased cholesterol uptake and a greater hepatic influx of cholesterol the portal blood are pathogenically relevant processes for humans that are causal to the observed accumulation of free cholesterol and liver inflammation. Although there is some associative evidence from an observational study that showed increased dietary cholesterol intake in NASH patients relative to controls (49), this does not demonstrate a causal link between cholesterol intake and hepatic inflammation in humans as such. Indeed, a controlled human intervention study by Tannock et al. (50) showed no evidence for an inflammation-inducing effect of dietary cholesterol in insulin-resistant subjects (i.e., the patient group that is at increased risk for NAFLD/NASH). Despite a strongly increased dietary cholesterol intake, there was no increase in plasma SAA or CRP levels, the latter being the most sensitive liver-specific inflammation marker available. Hence, the available evidence does not support the concept that dietary cholesterol, at relevant doses achieved in human diets, is an inducer of liver inflammation in humans. Rodent models that rely strongly on supraphysiological cholesterol supplementation most likely specifically allow investigation of direct hepatotoxic effects of dietary cholesterol. In the current study, we used a more comprehensive model for NAFLD that makes use of more translational dietary conditions and may better reflect the multifactorial and multi-organ nature of the disease (e.g., with an obese and insulin-resistant phenotype, with the involvement of adipose tissue hypertrophy and inflammation and increased gut permeability). We show here that hepatic cholesterol homeostasis is also deregulated under these more translational experimental conditions with realistic dietary cholesterol concentrations, leading to free cholesterol accumulation which contributes to the hepatic inflammatory response.

Given the multifactorial aetiology of NAFLD that involves deregulation of a multitude of pathways as drivers of disease progression, it seems appropriate to use a multitarget approach to combat the disease. Such an approach is increasingly considered the new paradigm for drug development for NAFLD, with numerous combination therapies currently under investigation (51–53). Here we used a multicomponent therapy with natural ingredients (HC-24) to attenuate NAFLD development, and studied its effects on several organs considered to be involved in the pathogenesis, i.e., adipose tissue (54, 55) and gut (permeability) (56, 57) in addition to the liver. We show here that HC-24 did not affect adipose tissue mass (measured as total body fat percentage as well as by the weight of several individual adipose tissue depots), adipocyte hypertrophy or adipose tissue inflammation in two visceral white adipose tissue depots (the epididymal and the mesenteric depots), and that disease-associated increased gut permeability was also not affected by HC-24 treatment – together indicating that the NASH-attenuating effect of HC-24 is most likely localised to the liver specifically rather than (partly) attributable to indirect effects via the adipose tissue or the gut.

In the liver, we observed a pronounced reduction in lobular inflammation in animals treated with HC-24. This reduction in inflammatory aggregates was associated with a decrease in the accumulation of free cholesterol in the liver which corresponded with the observed reduction in whole-body cholesterol production. A subsequent correlation analysis revealed that the number of inflammatory aggregates in the liver was strongly and significantly correlated with hepatic free cholesterol content, thus providing a potential explanation for the observed beneficial effects of HC-24 in NAFLD. These findings are in line with an earlier rodent study that showed a positive correlation between hepatic free cholesterol concentrations and hepatic NFκB activation, both of which were reduced after treatment with an anthocyanin-rich plant extract (58). An orchestrated adaptation of cholesterol biosynthesis and metabolism pathways and connected inflammatory pathways in both the liver and the adipose tissue was also observed in another study in response to a high-fat diet with a natural cholesterol content (59).

Besides these effects on transcriptional control mechanisms, another proposed mechanism by which cholesterol induces inflammation is by induction of physical cellular damage [for instance through formation of cholesterol crystals (60)] – which can result in the release of damage-associated molecular patterns (DAMPs) that function as a chemotactic signal for neutrophils (61) and may thus underlie the observed effect on neutrophil infiltration. Additionally, cholesterol loading in macrophages has been shown to induce expression of IL-8 – a potent neutrophil chemoattractant (62) – and cholesterol enrichment in neutrophil membranes has been found to enhance neutrophil adhesion and arrest, thereby increasing their potential for extravasation (63). In line with this, we found that the neutrophil chemokine CXCL1 was increased in livers of HFD control mice. This corresponds with observations in human NASH livers, which show a strong upregulation of CXCL1 that is not observed in livers from patients with simple steatosis (64, 65). Of note, other HFD-fed mouse models of NASH do not show this upregulation of CXCL1 (65, 66). A recent report by Hwang et al. (65) showed that overexpression of *Cxcl1* in HFD-fed mice was sufficient to induce NASH (recapitulating the pathological features of human NASH), thus further underlining the importance of this chemokine. Treatment with HC-24 significantly reduced CXCL1 expression in the liver — both on the gene expression level and on the protein level — and reduced infiltration of neutrophils into the liver. Although the infiltration of neutrophils is recognised as a defining characteristic of human NASH (67), their precise role in the pathogenesis of NASH has not been completely elucidated. However, their ability to release a potent cocktail of reactive oxygen species and proteases is thought to make them a potential cause of extensive tissue damage as demonstrated in other liver diseases (68, 69). This notion is further substantiated by the above-mentioned study on CXCL1-induced NASH (65), which provides several lines of evidence that neutrophils promote NASH development through production of ROS that activate several stress kinases (ASK1, p38/JNK-CASP3 pathway) in the liver, resulting in liver injury, inflammation and fibrosis. Conversely, depletion of neutrophils in HFD-fed mice reduces development of NASH (70). Since hepatic inflammation in the current study showed a clear correlation with hepatic free cholesterol levels, it is likely that the observed anti-inflammatory effects of HC-24 are (at least in part) secondary to the improvement of cholesterol homeostasis observed in HC-24-treated animals.

Overall, our results show that free cholesterol accumulates in the liver of Ldlr-/-.Leiden mice under physiologically translational dietary conditions and that this build-up of free cholesterol is associated with the development of hepatic inflammation. Treatment with the multicomponent medicinal product HC-24 reduces this free cholesterol accumulation and has molecular and cellular anti-inflammatory effects in the liver.

## Data Availability Statement

The data has been uploaded to the GEO database, under accession number GSE163652.

## Ethics Statement

The animal study was reviewed and approved by Animal Welfare Body (IvD) TNO the Netherlands (approval number 3682/TNO-210).

## Author Contributions

RK, YB, BS, and MM contributed to conception and design of the study. EG, WD, and AMe collected the data. LV, MC, KS, and MM analyzed the data. AMu, RK, NK, YB, BS, and MM interpreted the data. AMu, RK, and MM wrote the manuscript. All authors contributed to the article and approved the submitted version.

## Funding

This study was performed within the public-private partnership (PPP) ProLiver, a collaboration project that is co-funded by a PPP Allowance made available by Health~Holland, Top Sector Life Sciences & Health, to stimulate public-private partnerships. Heel GmbH was one of the private partners that contributed funding to the PPP ProLiver. The work described here was also supported by the TNO Research Programs Food and Nutrition and Biomedical Health.

## Conflict of Interest

AMu and BS are employees of Heel GmbH. NK and YB are former employees of Heel GmbH. Heel GmbH was involved in the design of the study and the preparation of the manuscript. Heel GmbH was not involved in data acquisition or data analysis. The publication of this study was a requirement of the funding received from Health~Holland, Top Sector Life Sciences & Health.

The remaining authors declare that the research was conducted in the absence of any commercial or financial relationships that could be construed as a potential conflict of interest.
