# Supplementary material for: Cholesterol Accumulation as a Driver of Hepatic Inflammation Under Translational Dietary Conditions Can Be Attenuated by a Multicomponent Medicine
Source: Front Endocrinol (Lausanne). 2021 Mar 18;12:601160. doi: 10.3389/fendo.2021.601160 (PMC8014004; doi:10.3389/fendo.2021.601160)
Supplement: Supplementary file 1 [file Table_1.docx]

**Supplemental table 1. Composition of HC-24**

| **Component** | | **Manufacturing method (Ph. Eur.)** | **µg/ml in HC-24** |
| --- | --- | --- | --- |
| *Plant extracts* | | | |
|  | *Avena sativa L.* (Oat), fresh aerial parts | Method 1.1.1 | 0,02 |
|  | *Chelidonium majus L.* (Greater celandine), fresh rhizome and adherent roots | Method 1.1.5 | 0,3 |
|  | *Cinchona pubescens* (Cinchona), dried bark | Method 1.1.8 | 10 |
|  | *Cynara scolymus L.* (Artichoke), fresh epigeal flowering parts | Method 1.1.5 | 0,03 |
|  | *Lycopodium clavatum L.* (Club moss), dried ripe spores | Method 1.1.8 | 10 |
|  | *Silybum marianum* (Milk thistle), mature fruit devoid of the pappus of the fruit | Ph. Eur 8.0/1860 | 10 |
|  | *Taraxacum officinale* (Dandelion), whole fresh flowering plants | Method 1.1.3 | 2 |
|  | *Veratrum album L* (White hellebore), dried rhizome and adherent roots | Method 1.1.8 | 10 |
| *Chemical substances* | | | |
|  | α-ketoglutaric acid | Method 3.1.1 | 1E-6 |
|  | Fumaric acid | Method 3.1.1 | 1E-6 |
|  | Malic acid | Method 3.1.1 | 1E-6 |
|  | Orotic Acid Monohydrate | Method 4.1.1 | 0,01 |
|  | Thioctic acid | Method 4.1.1 | 1E-4 |
|  | Calcium carbonicum Hahnemanni (inner parts of broken shells of the oyster) | Method 4.1.1 | 1E-24 |
|  | Cyanocobalamin (vitamin B12) | Method 3.1.1 | 1 |
|  | Histamin | Method 3.1.1 | 1E-6 |
|  | Diethyloxalacetate, sodium | Method 4.1.1 | 1E-6 |
|  | Sulphur | Method 4.1.1 | 1E-9 |
| *Extracts of organs from healthy pigs* | | | |
|  | Liver | Method 2.1.1 | 1E-4 |
|  | Colon | Method 2.1.1 | 1E-6 |
|  | Duodenum | Method 2.1.1 | 1E-6 |
|  | Pancreas | Method 2.1.1 | 1E-6 |
|  | Thymus | Method 2.1.1 | 1E-6 |
|  | Gall bladder | Method 2.1.1 | 1E-6 |

Ph. Eur. = European Pharmacopoeia
