# Supplementary material for: Cholesterol Accumulation as a Driver of Hepatic Inflammation Under Translational Dietary Conditions Can Be Attenuated by a Multicomponent Medicine
Source: Front Endocrinol (Lausanne). 2021 Mar 18;12:601160. doi: 10.3389/fendo.2021.601160 (PMC8014004; doi:10.3389/fendo.2021.601160)
Supplement: Supplementary file 3 [file Table_5.docx]

**Supplemental table 5. Bile acid and neutral sterol excretion and cholesterol intake at t=16 weeks and t=24 weeks**

|  | | **t=16 weeks** | | |  | **t=24 weeks** | | |
| --- | --- | --- | --- | --- | --- | --- | --- | --- |
|  | | **chow** | **HFD + vehicle** | **HFD + HC-24** |  | **chow** | **HFD + vehicle** | **HFD + HC-24** |
| **Total bile acid excretion** | | 1.46 ± 0.34 | 1.53 ± 0.16 | 0.92 ± 0.12 |  | 1.72 ± 0.32 | 2.25 ± 0.24 | 1.6 ± 0.26 |
|  | cholic acid excretion | 0.10 ± 0.04 | 0.07 ± 0.03 | 0.03 ± 0.01 |  | 0.06 ± 0.01 | 0.11 ± 0.03 | 0.10 ± 0.03 |
|  | deoxycholic acid excretion | 0.71 ± 0.21 | 0.92 ± 0.09 | 0.54 ± 0.11 |  | 0.89 ± 0.23 | 1 ± 0.13 | 0.68 ± 0.16 |
|  | litocholic acid excretion | 0.068 ± 0.019 | 0.017 ± 0.004 | 0.015 ± 0.003 |  | 0.057 ± 0.014 | 0.087 ± 0.017 | 0.044 ± 0.009 |
|  | β-muricholic acid excretion | 0.15 ± 0.04 | 0.18 ± 0.01 | 0.12 ± 0.01 |  | 0.23 ± 0.05 | 0.34 ± 0.04 | 0.24 ± 0.04 |
|  | ω-muricholic acid excretion | 0.24 ± 0.05 | 0.24 ± 0.02 | 0.16 ± 0.01 |  | 0.39 ± 0.03* | 0.61 ± 0.06 | 0.47 ± 0.04 |
|  | hyodeoxycholic acid excretion | 0.13 ± 0.01* | 0.09 ± 0.01 | 0.05 ± 0.01* |  | 0.09 ± 0.01 | 0.1 ± 0.01 | 0.06 ± 0.01 |
| **Total neutral sterol excretion** | | 2.09 ± 0.06* | 0.75 ± 0.11 | 0.82 ± 0.19 |  | 2.25 ± 0.22 | 1.59 ± 0.81 | 0.93 ± 0.22 |
|  | coprostanol excretion | 0.019 ± 0.003* | 0.003 ± 0.001 | 0.003 ± 0.001 |  | 0.031 ± 0.004 | 0.014 ± 0.006 | 0.008 ± 0.001 |
|  | cholesterol excretion | 1.87 ± 0.05* | 0.61 ± 0.09 | 0.69 ± 0.17 |  | 2.01 ± 0.21 | 1.32 ± 0.7 | 0.78 ± 0.20 |
|  | cholestanol excretion | 0.105 ± 0.007 | 0.115 ± 0.012 | 0.093 ± 0.01 |  | 0.105 ± 0.009 | 0.142 ± 0.021 | 0.096 ± 0.007 |
|  | lathosterol excretion | 0.094 ± 0.009* | 0.022 ± 0.005 | 0.035 ± 0.012 |  | 0.109 ± 0.02 | 0.113 ± 0.089 | 0.04 ± 0.014 |
| **Cholesterol intake** | |  | 0.74 ± 0.01 | 0.77 ± 0.02 |  |  | 0.86 ± 0.04 | 0.89 ± 0.04 |

Bile acid and neutral sterol excretion are expressed as µmol/mouse/day. Cholesterol intake is expressed as µmol/mouse/day. * p < 0.05 vs HFD + vehicle at the same timepoint.
